# Pathogen-Specific Circulating Plasmablasts in Patients with Pneumonia

**DOI:** 10.1371/journal.pone.0034334

**Published:** 2012-03-27

**Authors:** Nina V. Palkola, Sari H. Pakkanen, Jussi M. Kantele, Niina Rossi, Ritvaleena Puohiniemi, Anu Kantele

**Affiliations:** 1 Department of Bacteriology and Immunology, Haartman Institute, University of Helsinki, Helsinki, Finland; 2 Department of Medicine, Division of Infectious Diseases, Helsinki University Central Hospital, Helsinki, Finland; 3 Department of Medical Microbiology and Immunology, University of Turku, Turku, Finland; 4 Department of Bacteriology, Helsinki University Hospital Laboratory (HUSLAB), Helsinki, Finland; 5 Department of Medicine, Institute of Clinical Medicine, University of Helsinki, Helsinki, Finland; University of Cape Town, South Africa

## Abstract

Lower respiratory tract infections (LRTI) are the leading cause of death world-wide, with *Streptococcus pneumoniae* (Pnc) as the most prevalent pathogen. Local immune mechanisms appear central to protection against the disease, yet they are poorly characterized. Infections at other, non-respiratory mucosal sites are associated with a transient circulation of mucosa-originating lymphocytes from the mucosal site to blood and back to the mucosa. The present study explored whether pathogen-specific plasmablasts appear in the circulation also in patients with infection of the lower respiratory tract. 16 patients with bacteremic Pnc pneumonia and 14 healthy volunteers were explored for circulating plasmablasts secreting antibodies against their own pathogenic Pnc strain isolated in blood cultures (patients) or against several pathogenic strains from pneumonia patients (14 controls) or a mixture of nine different purified pneumococcal polysaccharides (8 controls). Both patients and volunteers were studied for all plasmablasts. The cells were identified with ELISPOT as Pnc-specific antibody-secreting cells (ASC) and as all immunoglobulin-secreting cells (ISC). High numbers of circulating Pnc-specific ASC were found in the acute phase of the disease in all patients with pneumonia (median 97 ASC/10^6^ PBMC), but in none of the controls. IgG isotype predominated in 9/16 patients. The numbers of ISC were significantly higher in the patients than in the healthy controls, yet Pnc-specific ASC only accounted for 0.7% of all the patients' ISC.The present study is the first to show that antigen-specific plasmablasts appear in the circulation in pneumonia, suggesting that pulmonary lypmhocytes recirculate in humans. Assessing these cells provides a novel tool for studying immune response to antigens encountered at the LRT.

## Introduction

The mucosa of the respiratory tract is constantly exposed to a vast variety of inhaled microbes, some of which may bring on disease. Lower respiratory tract infections (LRTI) are one of the leading causes of death world-wide [1], with *Streptococcus pneumoniae* (Pnc) as the most prevalent pathogen [2]. Colonization of the upper respiratory tract by Pnc is considered a significant preliminary step in the course of pneumonia [3]–[6]. Therefore, induction of local immune response preventing colonization appears beneficial in preventing pneumonia [3], [5], [6].

Even though the local immune mechanisms are considered essential in the immune defence in the respiratory tract [6]–[9], the mucosal immune mechanisms at the lower respiratory tract (LRT) are scantily characterized. Bronchus-associated lymphoid tissue (BALT) consists of discrete lymphoid aggregates in the bronchial mucosa. Like the gut-associated lymphoid tissue (GALT), BALT contains T and B cells, dendritic cells, macrophages and high endothelial venules [8], [10]. BALT and intestinal mucosa-associated Peyer's patches (PP) show many morphological and functional similarities; both BALT and PP provide entry for the mucosal pathogens through special epithelial cells (M cells), for example, and both are involved in the local production of IgA [8], [10], the main Ig-isotype at most mucosal sites [11]–[14]. However, differences have also been noted between BALT and PP: *in vitro* lymphocyte/endothelial binding, for example, suggests that BALT and PPs differ in their lymphocyte-binding selectivity [15], and IgG appears to be more significant in the LRT than the intestine [5], [16].

Probably due to practical and ethical restrictions in sampling at LRT in humans, the immune mechanisms at this site are not as well studied as the local system in the intestine. However, with other not easily accessible mucosal sites, such as the intestine [17]–[20], and the urinary tract [21], [22], it has proven possible to assess mucosal immune response using samples of peripheral blood. This approach is based on the recirculation of activated lymphocytes: antigen encounter at a mucosal site is followed by a recirculation of activated lymphocytes via lymphatics and blood back to mucosal sites [17]–[19], [21], [23], [24], where they are responsible for local antibody production [25]–[27]. The mucosa-originating antigen-specific plasmablasts (pre-plasma cells) can be caught from the circulation before they home to mucosal sites, and identified as pathogen-specific plasmablasts [17]–[20], [22]. In addition to homing to the site where the antigen activation took place, some of the cells appear to home to some other mucosal sites as well [28]. In this way the different mucosal sites within the mucosa-associated lymphoid tissues (MALT) are regarded to communicate with each other with help of migrating lymphocytes [7], [29]. This circulation of activated cells has been suggested to happen also at the lower respiratory tract: as early as 1980 it was shown in animal experiments that lymphocytes from PP and BALT have an equal propensity to repopulate mucosal tissues with IgA-plasmablasts [30]. Later, it was established in mice that adoptively transferred influenza-specific T cell clones can be relocated in the lung [31]. Until now, there have been no studies exploring whether antigen-specific plasmablasts appear in the circulation following antigen encounter at the LRT in humans. By showing an emergence of antigen-specific plasmablasts in the circulation during pneumonia, the present study not only provides evidence of a recirculation of pulmonary lymphocytes, but also presents a less invasive tool for future research into immune responses elicited at the LRT.

## Results

### Microbiological analyses

Throat cultures of the eight volunteers tested for pneumococcal carriage proved all negative. Data on serotype testing was available for 10/16 pneumococcal strains isolated in the blood cultures of the pneumonia patients (strains had been tested in a reference laboratorium in the National Institute for Health and Welfare, Helsinki). Five of them had serotype 14, the others serotype 4, 6B, 7F, 9V and 23F, each in one case.

### ASC response in patients with pneumonia

**Figure 1:**
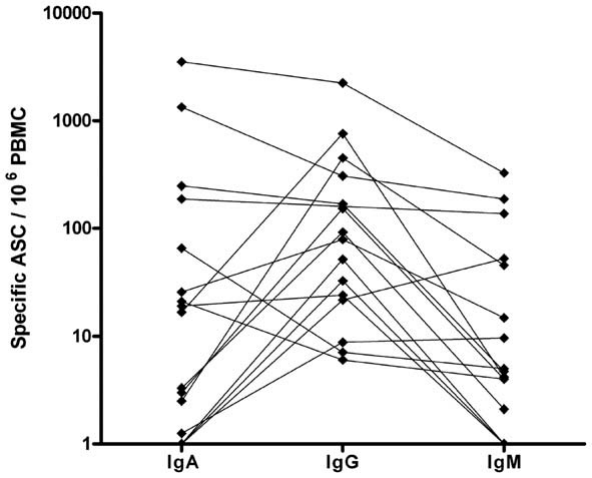
Plasmablast response in pneumococcal pneumonia. Numbers of pathogen-specific plasmablasts identified as antibody-secreting cells/10^6^ PBMC in IgA-, IgG- and IgM-isotypes in 16 patients with acute *Streptococcus pneumoniae* pneumonia. The IgA, IgG and IgM values of each individual volunteer are connected with a line.

No Pnc-specific ASC were found in the circulation of any of the healthy volunteers regardless of whether pneumococcal strains of pneumonia patients (two strains both tested in six volunteers, eight strains each tested in eight volunteers) or a mixture of nine capsular polysaccharides (tested in eight volunteers) was used as antigen. In contrast, all patients with pneumonia exhibited circulating ASC specific to their own Pnc strain on day 7–10 after the onset of symptoms (Figure 1).

**Figure 2:**
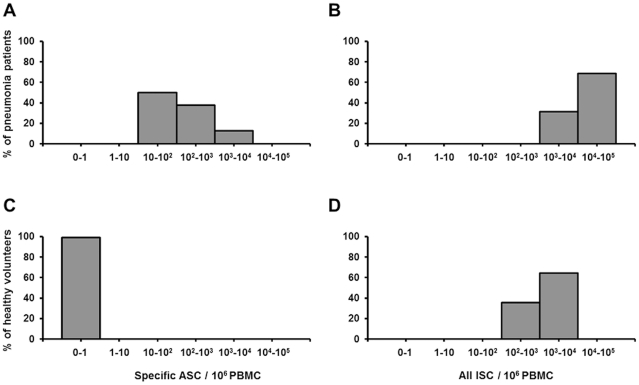
Levels of Pnc-specific plasmablasts and all plasmablasts in patients and controls. Percentage of pneumonia patients (n = 16) with indicated number of pathogen-specific ASC (A) and all ISC (immunoglobulin-secreting cells) (B) and percentage of healthy volunteers with indicated number of pathogen-specific ASC (C) and all ISC (D). ASC and ISC numbers are both presented as sum of IgA-, IgG- and IgM-secreting cells.

**Figure 3:**
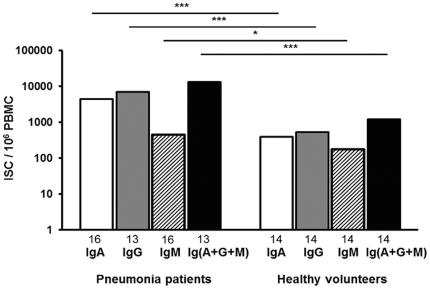
The numbers of ISC in patients with pneumonia and in healthy volunteers. The bars indicate medians of all ISC in IgA-, IgG- and IgM-isotypes. The statistical analysis was performed with independent samples t-test for IgG-ISC and with Mann Whitney U for the others. Statistical significances in comparison to numbers of ISC in healthy volunteers are indicated by asterisks (*** p<0.001; * 0.01<p<0.05). The numbers of volunteers from whom the data were pooled are indicated under each bar.

In patients with pneumonia, the median of the total number of pathogen-specific ASC (IgA+IgG+IgM) was 97/10^6^ PBMC (min-max 21–6112). There were eight moderate responders (10–100 ASC/10^6^), six high responders (100–1000 ASC/10^6^) and two very high responders (1,000–10,000 ASC/10^6^) (Figure 2). The medians of IgA-, IgG- and IgM-ASC are shown in Figure 3. The predominating isotype was IgA in 5/16, IgG in 9/16 and IgM in 2/16 patients (Figure 1). In a representative case, a comparison between ELISPOT assays using the patient's own Pnc-strain or a mixture of Pnc polysaccharide antigens revealed a significant response in both assays. The mixture of polysaccharide antigens did not prove any inferior to the patient's own strain (52 against own Pnc strain vs. 73 ASC/10^6^ PBMC against mixture of capsular polysaccharides).

### All ISC in patients with pneumonia and in healthy controls

IgG-ISC among patients and volunteers were found to be normally distributed while IgA-and IgM-ISC and the sum (IgA+IgG+IgM)-ISC were not. In patients with pneumonia, the median of the number of all ISC (IgA+IgG+IgM) was 13,181/10^6^ PBMC (min – max 3630–48,773) (Figure 3). This was significantly higher than the number of ISC in healthy controls (median 1,193/10^6^ PBMC; min - max 383–3,000, p<0.001 with Mann Whitney U test). The numbers of all ISC was higher in patients than in controls even when the numbers of Pnc-specific ASC were subtracted from the numbers of all patient ISC (median of “all patient ISC minus specific ASC” was 10,761/10^6^ PBMC; min - max 3,552–42,661; comparison to all control ISC p<0.001 with Mann Whitney U test). The numbers of Pnc-specific ASC represented only 0.7% of ISC in patients with pneumonia.

## Discussion

Mucosal exposure to *S. pneumoniae* induces both mucosal and systemic humoral and cellular immune responses [6], all considered to contribute to the protection against pneumonia. Recurrences of Pnc pneumonia rarely occur in immunocompetent patients [32]–[34], which suggests that the disease would induce host immunity against recurrent infections. Accordingly, it appears beneficial with Pnc vaccines, too, to induce responses mimicking natural responses. Because of practical and ethical restrictions on sampling at LRT in humans, however, the immune mechanisms in a natural infection are poorly characterized. For studying them, less invasive means are needed. A practicable option could be the identification of BALT-originating plasmablasts in the circulation of patients after antigen encounter at the LRT.

The present study is the first to show that pathogen-specific plasmablasts appear in the circulation in LRTI in humans. This finding, novel for LRTI, accords with previous studies, showing a transient appearance of circulating pathogen-specific plasmablasts in infections located at other human mucosal sites, for example bacterial gastroenteritis [18], [19], [35], urinary tract infection [21], [22], and upper respiratory tract infection [36], and, respectively, after mucosal vaccinations, such as oral [17], [20], [23], rectal [28] and intranasal [37] vaccinations. These cells are considered to represent lymphocytes that after antigen encounter recirculate from one mucosal site to the same and some others within the MALT, which explains why mucosal immunization at one site can lead to immune response at a distant site [8], [28]. The current finding of pathogen-specific plasmablasts in patients with pneumonia not only suggests that mucosal cells from the LRT migrate in humans, but also provides evidence that BALT participates in the lymphocyte-based communication between the different sites of the MALT.

So as to ensure that we would measure plasmablasts specific to the causative microbe of the pneumonia in each patient, we opted for investigating the response to each patient's own Pnc isolate, and only bacteremic patients were enrolled. Thus, even if the first encounter with the pathogen took place in the respiratory tract, because of the bacteremia some systemic stimulation was also likely to appear. The bacteremic period appears very short in comparison with the infection at the LTR and, therefore, a major immunological impact of the bacteremic condition is unlikely. This accords with the results of our previous study where pathogen-specific ASC were found both in bacteremic and nonbacteremic patients with a clinical picture and urine findings consistent with pyelonephritis [21].

Interestingly, in the present study, IgG predominated in 9/16 and IgA in 5/16 cases. The IgG predominance could be interpreted as a systemic influence on the response. However, this finding also accords with previous studies indicating that IgG per se may have a more pronounced defense role than IgA in the LRT [5], [16], and with the fact that mucosal exposure to Pnc induces both systemic and mucosal immune responses [6]. These data attest to the possibly integrated induction of respiratory and systemic immunity suggested in a previous study of recurrent infections of human upper airways [38].

While the advantages of investigating local immune response in the LRT simply from blood samples are obvious, two specific points need to be taken into consideration when applying this approach. The first is the availability of the pathogens to be used as antigens. In bacteremia the pathogen can be recovered in blood culture, but in non-bacteremic patients a preselected antigen panel should be made available. To compare these alternatives, we studied one representative patient with both his own Pnc strain and a mixture of nine capsular polysaccharides of representative Pnc serotypes as antigens in the assay. The results suggest that both methods can be applied. The same mixture of common capsular polysaccharide types may be used for all patients, keeping in mind the limitation that only the serotypes chosen for the mixture are covered. The second point to be considered is the timing of the sampling, as pathogen-specific ASC are only expected to be found in the circulation transiently, i.e. for less than one week, before they home to their destination in the tissues. This timing is based on the kinetics of mucosa-originating plasmablast response in studies with oral vaccines: ASC have been shown to appear in the blood on day 3, peak around day 7, and be undetectable again on day 14 [23]. However, we have recently detected in a natural infection, in patients with gastroenteritis, that the pathogen's persistence at a mucosal site can be associated with a prolonged recruitment of pathogen-specific plasmablasts in the blood [35]. It remains to be confirmed whether the time window could also in studies of LRTI be extended beyond the days 7–10 used in the present study.

Another point to note in the immune response to Pnc pneumonia was the exceptionally high total number of all plasmablasts (ISC) in all our patients' circulation. The numbers by far exceeded those of ISC in healthy volunteers even if the increase caused by Pnc-specific plasmablasts was taken into account. A similar situation has been reported in other mucosal infections, such as bacterial gastroenteritis [18] and upper urinary tract infection [21], but not in lower urinary tract infection [21] or after oral typhoid vaccination [29]. It thus appears to be a feature of serious infections, possibly implying polyclonal stimulation [18], [21]. This conception is supported by our observation of plasmablasts reactive with an irrelevant antigen, trinitrophenyl in patients with gastroenteritis [18]. A polyclonal stimulation associated with serious infections could be part of the mechanisms of life-long immunity, where by-stander-stimulation has been suggested as a mechanism of keeping memory to unrelated antigens [39], [40].

In conclusion, pathogen-specific plasmablasts (ASC) appear in the circulation of patients with pneumonia, which provides evidence on a participatory role of the respiratory tract in the lymphocyte-based communication between the various sites of the MALT. The response is accompanied by a significant increase in the total numbers of plasmablasts (ISC), greater than what the Pnc-specific plasmablasts account for, thus suggesting a polyclonal stimulation. Recognizing the time limitations posed by the transient appearance of antigen-specific plasmablasts, a new tool for future investigations into immune response in lower respiratory tract infections is provided.

## Materials and Methods

Pathogen-specific ASC and all ISC were explored in samples of peripheral blood of patients with bacteremic Pnc-pneumonia and in healthy volunteers.

### Patients, healthy volunteers, vaccines and samples

This study protocol was in accordance with the ethical standards of the responsible regional committee on human experimentation and with the Helsinki Declaration of 1975 (as revised in 1983) and was approved by the ethics committees of the Department of Medicine in Helsinki University Central Hospital and the Central Hospital of Central Finland. Written informed consent was obtained from all patients and volunteers.

16 patients (eight women and eight men, aged 30–67 years) treated in Helsinki University Central Hospital or Central Hospital of Central Finland for acute pneumonia caused by *Streptococcus pneumoniae* and 14 healthy volunteers were enrolled. One patient in the pneumonia group had asthma, coronary heart disease and diabetes, one had chronic atrial fibrillation and two had coronary heart disease as permanent diagnoses. Two were smokers and one was very obese. The diagnosis of Pnc pneumonia was based on the following criteria: (1) clinical symptoms of acute pneumonia: onset of dyspnoea and/or cough within the last 10 days and fever ≥38°C (2) verified new pulmonary infiltrates by chest X-ray, (3) enhanced serum levels of C-reactive protein (>50 µg/ml) and (4) blood culture yielding *S. pneumoniae*. The group of healthy volunteers (nine women and five men, aged 18–50) had no underlying diseases; one of them had a history of community-acquired pneumonia several years earlier.

All volunteers provided one sample of venous blood and, in addition, eight controls provided a throat culture to identify a potential pneumococcal carrier status. In the pneumonia group, the blood sample was drawn 7–10 days after the onset of symptoms of respiratory tract infection. This timing was based on our previous studies on mucosal [17], [23] and parenteral [28] antigen administration indicating that ASC are found in the circulation only transiently with a peak around 7 days after antigen encounter. Peripheral blood mononuclear cells (PBMC) were isolated using Ficoll-Paque and analyzed with enzyme-linked immunospot assay (ELISPOT) for numbers of Pnc-specific ASC and for total numbers of immunoglobulin-secreting cells (ISC) of IgA, IgG and IgM isotypes.

### Antigen

For patients with pneumonia, each patient's own *S. pneumoniae*-strain isolated from blood culture was grown on chocolate agar plates, formalin-killed and used as antigen in the ELISPOT assay as described previously [18]. To explore a possible use of capsular polysaccharide as antigens in the assay, one representative patient with Pnc-pneumonia was analyzed both with a Pnc-specific ELISPOT assay using the patient's own Pnc-strain (serotype 14) and an assay with a mixture of purified capsular polysaccharides (10 µg/ml of each type 3, 4, 5, 6B, 7F, 8, 14, 19F, 23F; ATCC, Bethesda, Maryland). For healthy volunteers, the coating antigen was either this same mixture of nine polysaccharide strains (8 volunteers) or a single Pnc-strain isolated from pneumonia patients: 6 volunteers were tested for 2 strains (serotypes 9V and 14) and 8 volunteers for 8 strains (serotypes 3, 4, 5, 6B, 7F, 8, 14 and 23F).

### ELISPOT assay of specific ASC and ISC

The isolated PBMC were assayed for pathogen-specific ASC and all ISC using ELISPOT as described previously [23]. In brief, 96-well microtiter plate wells were coated for the antigen-specific ASC-assay with a whole-cell preparation of formalin-killed *S. pneumoniae* strain or with a mixture of nine different purified pneumococcal capsular polysaccharide types (see above). In the ISC-assay, the plates were coated with a capturing antibody against human IgA (rabbit anti-human IgA, Dako, Glostrup, Denmark), IgG (goat anti-human IgG, Sigma St. Louis, MO) and IgM (rabbit anti-human IgM; Dako). The wells were washed and blocked with 1% BSA-PBS. The cells were incubated in the wells for 2–3 hours, and antibodies secreted during this time were detected with alkaline phosphatase-conjugated goat anti-human IgA (Sigma), IgG (Sigma) or IgM. (Shouthern Biotech, Birmingham, USA). The substrate (5-bromo-4-chloro-3-indolyl phosphate p-toluidine salt; Sigma) was added in melted agarose. The spots were enumerated under a light microscope, and each spot was interpreted as a print of one antibody-secreting cell. In three pneumonia patients, the background in the IgG-ISC prohibited counting the exact values but allowed rough estimation of the level of IgG-ISC.

### Statistics

ASC/ISC were characterized using medians and range of values. The distribution of the data was tested with Shapiro-Wilk's test. If the data were found to be normally distributed, the statistical analyses were carried out with independent samples t-test and if not, Mann Whitney U test was used. Differences were considered significant only when p<0,05. Statistical analyses were carried out using SAS system for Windows, Version 9.2 (SAS Institute Inc, Cary, NC, USA).
